# HIV LTR-Driven Antisense RNA by Itself Has Regulatory Function and May Curtail Virus Reactivation From Latency

**DOI:** 10.3389/fmicb.2018.01066

**Published:** 2018-05-25

**Authors:** Mie Kobayashi-Ishihara, Kazutaka Terahara, Javier P. Martinez, Makoto Yamagishi, Ryutaro Iwabuchi, Christian Brander, Manabu Ato, Toshiki Watanabe, Andreas Meyerhans, Yasuko Tsunetsugu-Yokota

**Affiliations:** ^1^Department of Immunology, National Institute of Infectious Diseases, Tokyo, Japan; ^2^Infection Biology Group, Department of Experimental and Health Sciences, Universitat Pompeu Fabra, Barcelona, Spain; ^3^Graduate School of Frontier Sciences, The University of Tokyo, Tokyo, Japan; ^4^Department of Life Science and Medical Bioscience, Waseda University, Tokyo, Japan; ^5^IrsiCaixa – AIDS Research Institute, Badalona, Spain; ^6^Universitat de Vic-Universitat Central de Catalunya, Vic, Spain; ^7^Institució Catalana de Recerca i Estudis Avançats, Barcelona, Spain; ^8^Department of Mycobacteriology, Leprosy Research Center, National Institute of Infectious Diseases, Tokyo, Japan; ^9^Department of Advanced Medical Innovation, St. Marianna University School of Medicine, Kawasaki, Japan; ^10^Department of Medical Technology, School of Human Sciences, Tokyo University of Technology, Tokyo, Japan

**Keywords:** HIV, viral antisense RNA, latency, reactivation, latency reversing agents

## Abstract

Latently infected T lymphocytes are an important barrier toward eliminating a persistent HIV infection. Here we describe an HIV-based recombinant fluorescent-lentivirus referred to as “rfl-HIV” that enables to analyze sense and antisense transcription by means of fluorescence reporter genes. This model virus exhibited similar transcriptional and functional properties of the antisense transcript as observed with a wild type HIV, and largely facilitated the generation of latently-infected T cells clones. We show that latently-infected cells can be divided into two types, those with and those without antisense transcription. Upon addition of latency reversal agents, only the cells that lack antisense transcripts are readily reactivated to transcribe HIV. Thus, antisense transcripts may exhibit a dominant suppressor activity and can lock an integrated provirus into a non-reactivatable state. These findings could have important implications for the development of strategies to eradicate HIV from infected individuals.

## Introduction

HIV latency is amongst the major obstacles to eradicate an established HIV infection (Richman et al., 2009). The current, highly efficient antiviral drugs are usually able to control HIV viremia to below detectable levels for extended time periods. However, therapy costs, drug resistance and long-term side effects are amongst the drawbacks that motivated research on HIV cure strategies (Walensky et al., 2006; Richman et al., 2009). These now comprise basic research of latency mechanisms and its reversion as well as augmenting and directing antiviral immune responses to HIV sanctuary sites by immunotherapeutic strategies (Deeks, 2012). Given that HIV reactivation from latency depends on provirus integration sites (Chen et al., 2017), the differentiation state of the infected cell (Lassen et al., 2012; Baxter et al., 2016; Tsunetsugu-Yokota et al., 2016), and inefficiency of with current latency reversal agents (Ho et al., 2013; Bullen et al., 2014), a further understanding about how HIV latency is generated, maintained and reverted is clearly needed.

HIV-encoded antisense transcripts may participate in latency (Kobayashi-Ishihara et al., 2012; Saayman et al., 2014; Zapata et al., 2017). The existence of antisense transcripts and encoded proteins have been first postulated by inspection of HIV sequences (Miller, 1988). Subsequent *in vitro* studies verified these postulations (Tagieva and Vaquero, 1997; Landry et al., 2007; Kobayashi-Ishihara et al., 2012) and demonstrated the existence of specific antibodies (Vanhee-Brossollet et al., 1995) and cytotoxic T cells (Bansal et al., 2010; Berger et al., 2015; Bet et al., 2015) against antisense-encoded proteins in HIV-infected patients, all suggesting that these sequences may have a functional role in the HIV life cycle *in vivo*. The main HIV antisense RNA named *ASP-L* is a 2.6 kb transcript. It encodes for ASP, a 189-amino acid long, hydrophobic protein that is rather conserved (Barbeau and Mesnard, 2015; Cassan et al., 2016; Dimonte, 2017). Although the function of ASP is poorly understood, an involvement in the enhancement of virus production from infected monocytes by stabilizing host autophagy machineries was suggested (Torresilla et al., 2013). We and other groups have previously shown that the antisense transcript itself, independent of an ASP coding function, can repress HIV-1 replication (Berkhout and van Wamel, 1995; Lu et al., 2004; Kobayashi-Ishihara et al., 2012; Saayman et al., 2014; Zapata et al., 2017). One possible mechanism is via its interaction with Polycomb Repressor Complex 2 (PRC2) that induces tri-methylation of lysine 27 on histone H3 (H3K27me3) at the 5′ LTR and thus gene silencing of HIV transcription (Saayman et al., 2014; Matsuda et al., 2015; Zapata et al., 2017). However, given that the expression level of antisense RNAs in bulk infected cells ranges from only 1/100 ∼ 1/2500 when compared to that of sense RNAs (Lefebvre et al., 2011; Kobayashi-Ishihara et al., 2012; Zapata et al., 2017), it is still unclear whether and under which conditions HIV antisense RNAs may influence virus latency. In the present study, we addressed this issue and analyzed the relation between antisense RNA expression and the latency status at the level of single infected cells. For this, a model lentivirus vector was constructed that enables to monitor HIV sense and antisense transcripts by fluorescent reporter gene expression. Using this system, we show that antisense transcript levels differ between latently-infected cell clones. Furthermore, in those clones with abundant antisense transcripts, sense transcription was hardly induced by T cell activation or latency-reversing agents suggesting that antisense transcription can exhibit a dominant inhibitory effect on virus reactivation.

## Materials and Methods

### Plasmid Preparation

For generating a model lentivirus vector, we first established pCSII-EF-MCS-IRES2-mCherry vector, to construct an expression system of mCherry gene by IRES-dependent translation (IRES-mCherry cassette). The mCherry gene fragment, obtained by digesting pmCherry (Clontech, Mountain View, CA, United States) with AgeI/SpeI, was replaced with the Venus gene of pCSII-EF-MCS-IRES2-Venus (kindly provided by Dr. Hiroyuki Miyoshi, BioResearch Center, Riken Tsukuba Institute, Tsukuba, Japan) by XbaI/BstXI digestion. The IRES-mCherry cassette was then isolated by EcoRI digestion from pCSII-EF-MCS-IRES2-mCherry. Second, the GFP gene of the Lenti LTR-GFP vector (Tsunetsugu-Yokota et al., 2016) was placed into the Venus gene in antisense direction by EcoRI/ KpnI. The generated lentiviral vector, pCSII-3′ LTR-Venus, harbors the Venus gene downstream of intact HIV-1 3′ LTR as well as Rev-responsive element (RRE), central polypurine tract (cPPT), and central termination sequence (CTS) downstream of HIV-1 5′ LTR. Third, the IRES-mCherry cassette was inserted into an EcoRI site of pCSII-3′ LTR-Venus in a sense orientation. Fourth, the Tat ORF, obtained by digesting pcDEBTat (kindly provided by Dr. Yutaka Takebe, NIID, Japan) with SacI/BamHI, was inserted upstream of the IRES-mCherry cassette by NotI/BamHI digestion. The final construct, pCSII-Tat-IRES-mCherry-3′ LTR-Venus vector, is referred to as *rfl-HIV* and was used in this study.

For propagation of pseudotyped lentiviruses, the packaging plasmids pCMV-VSV-G-RSV-Rev and pCAG-HIV*gag/pol* were provided by Dr. Hiroyuki Miyoshi. Viruses HIV-1 NL4-3 and HIV-1 NL-EΔenv were prepared from plasmids pNL4-3 (Adachi et al., 1986) and pNL-EΔenv (Tsunetsugu-Yokota et al., 2016) by transfection of 293T cells, respectively.

### Cells, Reagents and Extraction of DNA and RNA

HEK293T and CEM T cells were maintained in DMEM and RPMI 1640, respectively, supplemented with 10% heat-inactivated fetal bovine serum (FBS), 100 U/ml penicillin/streptomycin and 2 mM L-glutamine (Invitrogen, San Diego, CA, United States). Peripheral blood mononuclear cells (PBMCs) were isolated from healthy donors by Ficoll-Paque gradient centrifugation (Amersham Biosciences) and treated with 10 ng/ml PHA-P (Phytohemagglutinin-P, Sigma Aldrich, St. Louis, MO, United States) for 48 h. The activated PBMCs (PHA-blasts) were cultured in RPMI 1640 with 10% FBS, antibiotics and 20 U/mL of human recombinant IL-2 (R&D systems).

For latent rfl-HIV reactivation, PMA (phorbol 12-myristate 13-acetate, Sigma Aldrich) plus Ionomycin (Cayman Chemical, Ann Arbor, MI, United States), or SAHA (Vorinostat, Sigma Aldrich) were used at 20 ng/ml, 1 and 5 μM, respectively.

Cellular DNA and RNA were extracted by the NucleoSpin RNA kit with RNA/DNA buffer set (Machery-Nagel, Düren, Germany) according to the manufacturer’s protocol.

### Viral Productions and Infections

HIV-1 NL4-3, HIV NL-E_Δenv_ and rfl-HIV viruses were propagated as previously described (Yamamoto et al., 2009; Tsunetsugu-Yokota et al., 2016). In brief, HEK293T cells were transfected or co-transfected with respective plasmids by the calcium phosphate method (Conn et al., 1998). Four hours after transfection the culture supernatant was replaced with a fresh DMEM containing 10% FBS, L- glutamine, and antibiotics. Virus-containing supernatant was collected 48 h later, clarified by centrifugation (2500 rpm for 20 min at 4°C) and kept frozen at -80°C. Titrations of the supernatant were measured by TCID_50_ using MAGIC-5A cells (Hachiya et al., 2001) or an in-house HIV-1 Gag p24 enzyme-linked immunosorbent assay (ELISA) (Tsunetsugu-Yokota et al., 1995). According to the mCherry-expressing frequency in rfl-HIV-infected CEM cells, we determined that MOI = 1 was achieved by adding 1 μg of p24 to 10^6^ CEM cells.

To infect 2 × 10^7^ activated PBMC, 6 × 10^5^ TCID_50_ of HIV NL4-3 were used. After 24 h, cells were washed by PBS and cultured in a 10 cm dish. CEM T cells were infected with HIV-1 NL-EΔenv at MOI = 1. After 24 h incubation, cells were expanded with 10% FBS + RPMI for following sorting. For rfl-HIV infection, CEM T cells were either mock infected or infected with lentiviruses by spinoculation at room temperature, 1200 ×*g* for 2 h at MOI = 0.5. Cells were resuspended and cultured in 10% FBS+RPMI medium.

### Flow Cytometry

HIV NL-E_Δenv_ or rfl-HIV-infected CEM cells were stained with Live-dead Aqua (Invitrogen, San Diego, CA, United States) diluted in staining buffer (PBS with 2% FBS and 0.05% Sodium Azide) on ice 20 min, and analyzed or sorted by FACS Aria IIIu (BD Bioscience, Heidelberg, Germany). The acquired data was analyzed by Flowjo version 9.7.6 (Tree Star Inc., San Carlos, CA, United States).

### Strand-Specific Quantitative RT-PCR and Semi-Quantitative RT-PCR or PCR

Procedures of strand-specific quantitative (q) RT-PCR and semi-qRT-PCR were established in a previous study (Kobayashi-Ishihara et al., 2012). In brief, strand-specific cDNA was synthesized from total RNA with a RT primer harboring the tag sequence (Tag-RT primer) by SuperScript III Reverse Transcriptase (Invitrogen). Then the cDNA was amplified with Tag primer and a gene-specific primer using Thunderbird SYBR qPCR Mix (Toyobo, Osaka, Japan) or AccuPrime DNA polymerase (Invitrogen), respectively, for absolute and semi-quantifications. Absolute quantification was done with a standard curve drawn by a plasmid harboring a target amplicon.

Absolute quantification of HIV NL4-3 sense and antisense RNAs was done with following primer sets: For sense RNA quantification, 7743r tag primer was used for cDNA synthesis, then Tag primer and p5F primer was used in the PCR reaction. For antisense RNA quantification, p5F tag primer was used for cDNA synthesis, then Tag primer and 7443r primer were used in the PCR reaction. Sequences of these primers have been described elsewhere (Kobayashi-Ishihara et al., 2012).

For quantifying rfl-HIV sense and antisense transcripts, cDNAs were synthesized by Tag-mCherry R (5′-ctgatctagaggtaccggatcctcgttgtgggaggtgat-3′) and Tag-egfp2R (5′-ctgatctagaggtaccggatccgaactccagcaggaccatg-3′), respectively from 200 ng total RNA. Then, 1/10 of each cDNA product was separately followed by qPCR using Tag primer and mCherry F (5′-cactacgacgctgaggtcaa-3′) or egfp2F (5′-gaccactaccagcagaacac-3′), respectively. To measure the RNA level of RNase P as an internal control, cDNA was synthesized with random primer, then amplified with RNase P-F (5′-agatttggacctgcgagcg-3′) and RNase P-R (5′-gagcggctgtctccacaagt-3′) in a qPCR step (Tsunetsugu-Yokota et al., 2016).

Proviral load of unsorted cells or the EGFP-negative cell population infected with HIV NL-E_Δenv_ were analyzed by semi-qPCR using AccuPrime DNA polymerase with p5F and 7443r primers. According to a similarity of band density of the PCR products, we determined the proviral load of unsorted cells to be roughly three times higher than those of the EGFP-negative cell population. Therefore, 33 and 100 ng total RNAs, respectively, from unsorted and EGFP-negative cell populations were used for the following semi-quantification of viral transcripts. Semi-qRT-PCR were conducted using the same primer sets described in the quantification method for HIV NL4-3 sense and antisense RNAs.

### Transfection of Locked Nucleic Acid

Locked Nucleic Acid (LNA) was used to knockdown antisense RNAs transcribed from rfl-HIV. LNA targeting the Venus coding region of antisense RNA (5′-ctggtagctcaggtag-3′) and a negative control LNA (LNA GapmeR negative control B) were purchased from Exiqon (Vedbaek, Denmark). 300 pmol of each LNA was separately introduced into rfl-HIV-infected CEM cells by the Neon electroporation system (Invitrogen) using 1 pulse of 1230 V, 40 s. Transfected cells were maintained in RPMI with 10% FBS for 72 h.

### Sequence Analysis of rfl-HIV Proviruses and Alu-PCR

The gene body and the 5′ LTR sequence of rfl-HIV proviruses were amplified from cellular DNA and sequenced. To amplify the gene body of rfl-HIV (inner sequence between 5′ and 3′ LTRs), touchdown PCR was performed using KOD Fx DNA polymerase (Toyobo) with U5 F primer (5′-ccgtctgttgtgtgactctgg-3′) and p5R primer (5′-gctctagagcgagtgaattagcccttccagtc-3′). The PCR reaction conditions were 40 cycles of 98°C for 10 s, 60–52°C (0.2°C reduction every cycle) for 30 s and 68°C for 4 min after the 2 min pre-denaturation at 94°C. To amplify 5′ LTR, touchdown PCR was performed using KOD Plus DNA polymerase (Toyobo), with Gag R primer (5′-caatatcatacgccgagagtgcgcgcttcagcaag-3′) and 9076f primer (5′-ggtacctggaagggctaattcactccca-3′). The PCR reaction conditions were 40 cycles of 98°C for 15 s, 60-52°C (0.2°C reduction every cycle) for 30 s and 68°C for 1 min after the 2 min pre-denaturation at 94°C. The respective PCR products were gel-purified and subjected to Sanger sequencing by the FASMAC sequencing service (Kanagawa, Japan).

For quantifying integrated rfl-HIV proviral copies, Alu-PCR was conducted as previously described as integration assay (Tsunetsugu-Yokota et al., 2016).

### Statistical Analysis

For the statistical analysis, Prism software ver. 7 (GraphPad Software Inc., San Diego, CA, United States) was used. Student’s *t*-test or Mann–Whitney’s test were applied for evaluating statistical differences.

## Results

### HIV-Encoded Antisense RNA Is Transcribed in Latently Infected Cells

**FIGURE 1:**
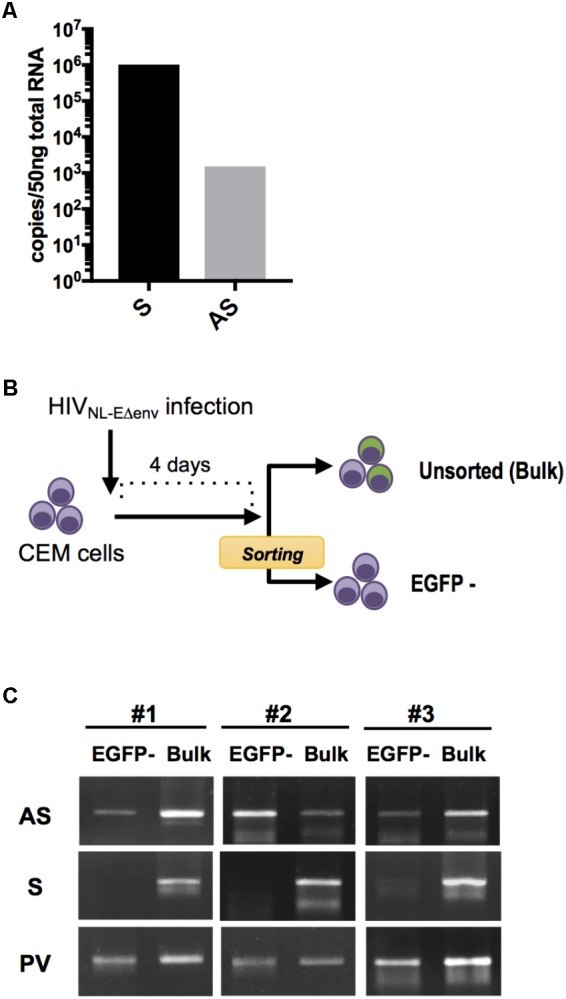
HIV sense and antisense transcript quantification within infected lymphocytes. **(A)** Absolute quantification of sense (S) and antisense (AS) transcripts within HIV NL4-3-infected PBMC at 7 dpi. Results from independent infection experiments using cells from 2 blood donors are shown as mean value. **(B)** Experimental strategy to analyze HIV transcripts in latently-infected cells. CEM cells were infected with pseudotyped HIV NL-E_Δenv_ that carries a EGFP reporter gene and sorted 4 dpi by flow cytometry to obtain a cell population that includes latently-infected (EGFP-negative) cells. DNA and RNA from the EGFP-negative as well as the bulk cell population were isolated and analyzed by semi-quantitative PCR and RT-PCR. **(C)** Levels of S and AS transcripts as well as HIV provirus DNA (PV) was analyzed in 3 independent experiments. Agarose gel images of PCR products are shown.

HIV-encoded antisense transcripts might be a viral latency factor exerting its activity at a low copy number per infected cell (Kobayashi-Ishihara et al., 2012; Saayman et al., 2014; Zapata et al., 2017). To first verify the ratio of sense and antisense transcripts in physiologically-relevant target cells, peripheral blood mononuclear cells (PBMCs) from two blood donors were stimulated with phytohaemagglutinin (PHA) and infected *in vitro* with HIV NL4-3. At 7 days post infection, cells were harvested, the total RNA was extracted and subjected to absolute quantification of HIV sense and antisense transcripts by a previously described quantitative, strand-specific RT-PCR procedure (Kobayashi-Ishihara et al., 2012). HIV sense transcripts were about 680 times more abundant than the antisense transcripts (**Figure 1A**), consistent with our previous observations using PBMCs (Lefebvre et al., 2011; Kobayashi-Ishihara et al., 2012; Zapata et al., 2017).

Next, we examined whether HIV antisense RNA is also transcribed in latently infected cells. CEM T cells were infected with VSV-pseudotyped HIV NL-E_Δenv_. This virus harbors a EGFP reporter gene in the sense orientation and thus allows to visualize a productive infection (Terahara et al., 2011). At 4 days post infection (dpi), EGFP-negative cells were isolated by flow cytometry (purity > 99%) (**Figure 1B**). Subsequently, unsorted cells and the EGFP-negative cell population that includes the latently infected cells were both subjected to total DNA and RNA extraction, and semi-quantitative PCR and RT-PCR for amplifying proviral genomes and viral transcripts, respectively (**Figure 1C**). HIV antisense transcripts were detected in both cell preparations while HIV sense transcripts were hardly observed in EGFP-negative cells but readily detected in unsorted cells. There was no amplification signal in uninfected control CEM T cells (data not shown). Thus, HIV antisense RNAs are transcribed in cells with latent as well as productive HIV infection. These results are compatible with the idea that antisense transcripts may be generated independently from transcriptional activity in the sense direction.

### Latently-Infected Cells Can Be Divided According to Their Antisense RNA Expression

**FIGURE 2:**
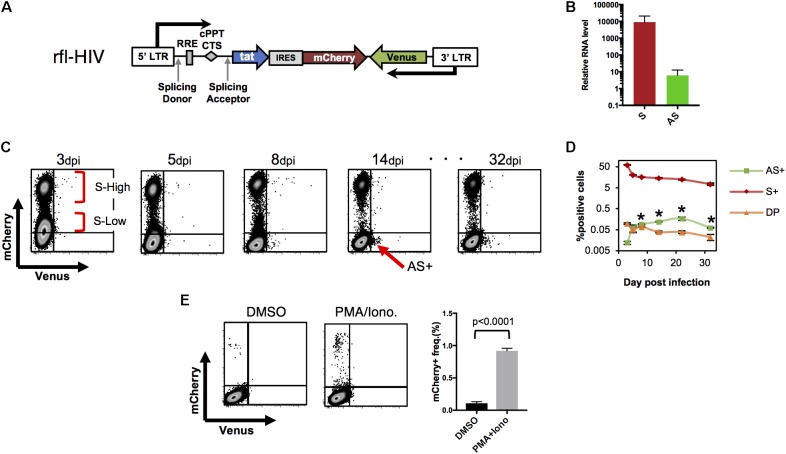
HIV-LTR driven fluorescent reporter virus with a wild-type HIV expression pattern. **(A)** Structure of rfl-HIV provirus. **(B)** Transcript levels of sense and antisense RNAs of rfl-HIV-infected CEM cells. RNAs were isolated 7–14 dpi and quantified by strand-specific RT-qPCR using primers within the mCherry and Venus coding region. Levels given are relative to RNase P RNA (*n* = 3, mean ± SD). **(C,D)** Expression of sense and antisense RNAs after rfl-HIV infection of CEM T cells. Cells were infected by pseudotyped rfl-HIV, followed by flow cytometry. Dot Plots for mCherry and Venus expression **(C)**, and the mean frequencies of mCherry (S+), Venus (AS+) and both (DP) positive cells are summarized (**D**; *n* = 3, mean ± SD). Each asterisk stands for a significant difference (*p* < 0.005) in the frequency between AS+ and DP cells at the same dpi. *P*-values were calculated by Student’s *t*-test. **(E)** Infected cells lacking mCherry and Venus expression contain latent rfl-HIV. Dot blot of rfl-HIV-infected CEM cells sorted at 7 dpi and kept in DMSO or PMA/Ionomycin for 24 h. Frequencies of mCherry expression are shown in the right panel (*n* = 3, mean ± SD). A *p*-value was calculated by Student’s *t*-test.

To study further the expression patterns of HIV sense and antisense RNAs in infected cells, we generated a HIV LTR–driven fluorescent reporter virus (recombinant fluorescent-HIV, rfl-HIV; **Figure 2A**). It carries a Venus gene in the antisense orientation downstream of the 3′ LTR promoter and a Tat – IRES – mCherry gene cassette in the sense orientation downstream of the 5′ LTR promoter. Rfl-HIV enables to monitor simultaneously sense and antisense transcription by the expression of mCherry and Venus, respectively, at a single cell level.

To first test whether the ratio between sense and antisense transcripts of rfl-HIV-infected CEM cells equals those of HIV-infected PBMCs, CEM T cells were transduced with rfl-HIV at a MOI of 0.5, total RNA was isolated, and sense and antisense transcripts were quantified by strand-specific RT-qPCR. The antisense RNA was about 800–1300 times less abundant than sense RNA (**Figure 2B**), in line with what we have observed for HIV-infected PBMCs. This indicated that rfl-HIV-infection of CEM cells shows similar transcription characteristics to wild type HIV-infection.

To investigate the expression of sense and antisense transcripts at a single cell level and to follow its evolution over time, rfl-HIV-infected CEM cells were analyzed by flow cytometry. mCherry single positive cells, Venus single positive cells, double positive cells and double negative cells were designated as S+, AS+, DP, and DN cells, respectively. Representative dot plots are shown in **Figure 2C** and the overall kinetics in **Figure 2D**. The cells at 3 dpi mainly contained S+ and DN. The S+ population contains two major populations, S-high-expressing (S-High) and S-low-expressing (S-Low) cells. In addition, a few antisense-expressing cells were observed in the DP population (around 0.1%). From 3–8 dpi, S-Low cells gradually disappeared while DN and AS+ cells appeared. After 14 dpi, the number of S-Low cells is low and S-High cells are the main S+ cell population. Thus, S-Low cells are apparently unstable and may have turned into latently or productively infected cells, a property that has been attributed to fluctuations in Tat expression (Weinberger et al., 2005). Interestingly, a small cell population appeared in the AS+ fraction (arrow in **Figure 2C**). This AS+ population was maintained until the end of the cell cultivation period at 32 dpi. Overall, the frequency of S+ and DP decreased sequentially after the first analysis at 3 dpi (**Figure 2D**). The frequency of AS+ cells increased and became significantly higher than that of DP cells at 8 dpi (asterisks in **Figure 2D**, *p* < 0.005). Collectively, the results from **Figures 2C,D** show the general characteristics of antisense expression: antisense RNA is preferentially expressed in productively infected cells (DP cells) at an early phase. When the frequency of sense-expressing cells decreases, the most of antisense-expressing cells are within latently-infected (mCherry-negative) cells.

To investigate whether latently infected cells also reside within the pool of DN cells, DN cells were sorted at 7 dpi by flow cytometry. They contained around 0.01 copies of integrated virus per cell as evidenced by Alu-PCR (data not shown). When treated with PMA/Ionomycin, latent rfl-HIV was readily reactivated (**Figure 2E**). Around 0.95% of DN cells produced mCherry demonstrating that this subpopulation of latently infected cells could turn into productive infection. Thus, latently infected cells are found within both, AS+ and DN cell populations.

### Antisense rfl-HIV Transcripts Inhibit Expression of Sense Transcripts

**FIGURE 3:**
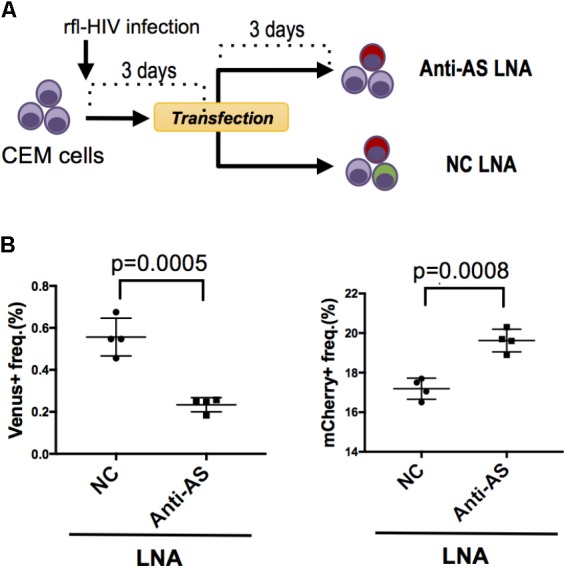
Knockdown of HIV antisense transcripts enhances HIV sense expression. **(A)** Outline of the experimental strategy. CEM cells were infected with rfl-HIV and transfected with either antisense transcript-specific LNAs (Anti-AS LNA) or negative control LNAs (NC LNA) at 3 dpi. Cells were analyzed by flow cytometry 72 h post transfection. **(B)** Knockdown of HIV antisense transcripts by Anti-AS LNAs reduces expression of the antisense RNA-encoded Venus (left panel) and enhances the sense-encoded mCherry genes (right panel). The frequencies of Venus- and mCherry-expressing cells are shown (*n* = 4, mean ± SD). *P*-values were calculated by Student’s *t*-tests.

We have previously shown that HIV antisense transcripts repress the expression of HIV sense transcripts (Kobayashi-Ishihara et al., 2012). To test whether rfl-HIV retains a similar function in its antisense RNA, we infected CEM cells with rfl-HIV and transfected these at 3 dpi with either a Locked Nucleic Acid (LNA) specific for the antisense RNA (Anti-AS LNA) or with a negative control LNA (NC LNA) (**Figure 3A**). The Anti-AS LNA reduced the frequency of Venus-expressing cells indicative of an efficient inhibition of antisense transcripts as expected (**Figure 3B**, left panel). Importantly, this inhibition of antisense transcripts led to an increase of sense transcript expression (**Figure 3B**, right panel) demonstrating that antisense RNA of rfl-HIV harbors a sense-repressive function similar to that of wild type HIV.

### Selection of rfl-HIV-Infected Cells Clones With Different Patterns of Sense and Antisense Transcription

**FIGURE 4:**
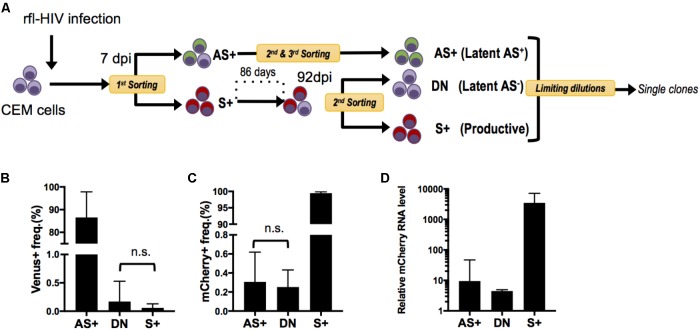
Generation and characterization of rfl-HIV-infected CEM cell clones. **(A)** Scheme of the experimental strategy. CEM cells were infected with rfl-HIV, cultured and subsequently separated into 3 different cell populations: sense transcript positive (=productive) cells (S+) and latently-infected cells that are either antisense transcript positive (Latent AS+) or antisense transcript negative (Latent AS–). These latter are abbreviated as AS+ and DN, respectively. In order to enrich Latent AS+ cells, several rounds of preparative flow cytometry were performed as indicated. **(B,C)** Basal expression frequency of antisense **(B)** and sense RNAs **(C)** in different cell clones. Shown are mean values of 3 individual cell clones each at 2 different time points (*n* = 6, mean ± SD). *P*-values were calculated by Mann–Whitney’s tests. **(D)** Relative levels of sense RNAs in each cell clone at one time point. Results of sense strand-specific RT-qPCR are shown (*n* = 3, mean ± SD). Primers were specific for the mCherry coding region.

Since rfl-HIV antisense RNA has a suppressive function on sense RNA expression, we speculated that this may also contribute to maintain HIV latency once established. However, as the populations of AS+ and DN cells may contain not only truly latently infected cells but also cells with aberrant and defective HIV proviruses, we generated infected cell clones and separately analyzed their sense and antisense HIV RNA expression. CEM cells were again infected by rfl-HIV, sorted into S+ and AS+ cell populations by flow cytometry, and isolated into single cells by limiting dilution (**Figure 4A**). Due to the very low frequency of AS+ cells, several steps of cell expansion and sorting were necessary to end up with a pure AS+ cell population for cloning. To obtain latently infected DN cells, the S+ cell population was expanded for around 3 months and then sorted into DN and S+ cells. Finally, from three pure cell populations, three individual cell clones were generated. Individual clones were then expanded to around 2 × 10^5^ cells, tested for provirus content by PCR and sequencing, and analyzed for their sense and antisense RNA expression by flow cytometry. Of the total of nine rfl-HIV-infected cell clones, the DN clone DN1H11 had a 300 bp deletion within the non-coding region of rfl-HIV provirus (Supplementary Figure S1), whereas all other clones were full length. Their individual transcript expression levels are shown in Supplementary Figure S2. The mean level for the grouped AS+, DN and S+ clones is shown in **Figures 4B,C**. The cells of the AS+ Venus-expressing clones AS+1D1, 2G8 and 10G18 as well as the DN clones DN12D7c, 12E21, and 1H11 were basically devoid of mCherry expression, while cells of the S+ mCherry-expressing clones S+2A1, 1D10 and 1C7 and the DN clones were devoid of Venus expression. These expression patterns were consistent with the levels of sense transcripts of all the clones (**Figure 4D**). Together, these results demonstrated that the expression features of the cell clones were stably maintained at least during the cultivation period of the cell clones and that the latently infected DN and AS+ clones did not spontaneously reactivate sense transcription.

### Antisense-Expressing Latently Infected Cells Are Refractory to Latency Reversal

**FIGURE 5:**
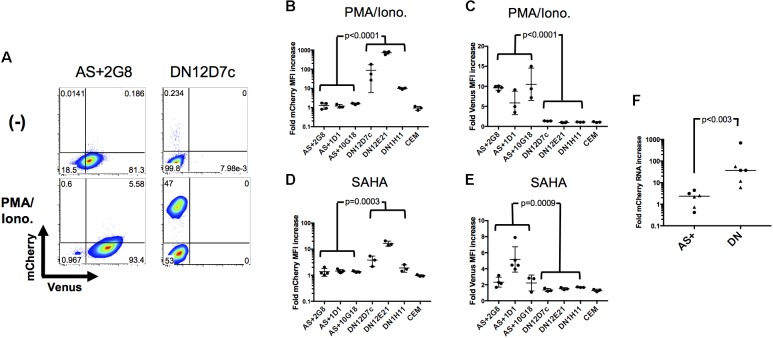
Sense RNA reactivation is influenced by antisense RNA transcription. **(A)** Representative results of sense (mCherry) and antisense RNA expression (Venus) after PMA/Ionomycin treatment of 2 individual cell clones (AS+2G8 and DN12D7c). Dot plots from flow cytometry at 24 h post stimulation are shown. **(B–E)** Increase of sense and antisense RNA expression in cloned cells after activation with PMA/Ionomycin **(B,C)** or SAHA **(D,E)**. The fold increase of the MFI of mCherry and Venus expression is shown for all individual cell clones. Fold increase was calculated from mCherry or Venus MFI of activated cells using that of non-activated (DMSO treated) cells as reference. Means ± SD of three to five independent experiments are given. *P*-values were calculated by Mann–Whitney’s tests. **(F)** Fold sense RNA increase after cell clone activation with PMA/Ionomycin or SAHA. Relative levels of mCherry RNA were measured by sense-strand-specific RT-qPCR. Dots represent PMA/Ionomycin-treated clones; triangles represent SAHA-treated clones. The p value was calculated by Mann–Whitney’s test.

In the above, two types of latently infected cells have been identified and cloned, those that express antisense transcripts (AS+ clones) and those that do not (DN clones). To investigate whether they would behave differently upon exposure to latency reversing agents and stimulation, they were treated with PMA/Ionomycin or the HDAC inhibitor SAHA (Vorinostat) and analyzed by flow cytometry. A representative example is shown in **Figure 5A** as a dot plot, and the results for all clones are given in **Figures 5B–E** as fold increase of mean fluorescence intensities (MFIs) for mCherry and Venus expression, respectively. A clear distinction between DN and AS+ clones was observed. Latent rfl-HIV was readily reactivated in the DN clones by both SAHA and PMA/Ionomycin as judged by an increase in the frequency of mCherry-expressing cells and an increase of their MFIs. T cell activation by PMA/Ionomycin was stronger than SAHA treatment with respect to the MFI increase. In contrast, neither stimulus resulted in virus reactivation of the AS+ clones. Rather increased expression of antisense transcripts was observed with both activating agents. Taken together, our results indicate that antisense expression in latently infected T cells is dominant over sense expression.

To demonstrate that these observations were ascribed to the effect at a transcriptional level, we quantified the respective level of sense RNAs after addition of latency reversal agents by strand-specific RT-qPCR and compared the difference between AS+ and DN clones (**Figure 5F**). The sense RNA levels increased significantly more in DN clones than in AS+ clones (*p* < 0.003) which is in concordance with the observed expression changes determined by flow cytometry. Furthermore, we excluded the possibility that the transcriptional differences between AS+ and DN proviruses were due to mutations in the 5′ LTR by sequencing (Supplementary Figure S3). Taken together, these results demonstrated that antisense RNA expressing latently infected cells can escape latency reversal.

## Discussion

The most intriguing finding of our work is the existence of 2 types of latently HIV-infected T lymphocytes, those that maintain antisense transcripts while lacking sense transcripts and those that are transcriptionally silent for both types of transcripts. When latency reversal agents were added to the respective cells, the former increased antisense but remained silent with respect to sense transcription. In contrast, the latter were readily reactivated. Together, these observations suggest that antisense transcription plays a role in the maintenance of HIV latency and that it marks cells that will not further contribute to virus spreading but maintain provirus that with current strategies can hardly be eradicated.

While the transcription of retroviruses in the sense direction gives rise to new viral genomes and the production of the main structural and regulatory proteins, antisense transcript-encoded proteins with roles in autophagy (ASP of HIV-1), and proliferation and latency (HBZ of Human T cell Leukemia Virus, HTLV-1) have been described (Gaudray et al., 2002; Satou et al., 2006; Arnold et al., 2008; Torresilla et al., 2013; Ma et al., 2016). Interestingly, the disruption of antisense protein production (Kobayashi-Ishihara et al., 2012; Zapata et al., 2017) as well as the exchange of the retrovirus-encoded sequence with a non-related fluorescence marker gene, as shown here, left a regulatory function of the antisense RNA itself intact. When antisense transcripts are produced, the transcription of sense RNA is reduced. Elegant work from Saayman and colleagues has shown that HIV antisense transcripts act like many long non-coding RNAs (lncRNAs) and direct epigenetic silencing components to the 5′ LTR shutting down sense transcription (Saayman et al., 2014). Since our rfl-HIV construct analyzed here and the complete HIV antisense transcript share only RNA sequences within the U3 region of the LTRs, this 376-base region might be of critical importance for these suppressive features. Indeed, this notion is consistent with our previous observations that an HIV antisense transcript lacking the U3 region also lost its suppressive function (Kobayashi-Ishihara et al., 2012).

The suppressive function described above may be kept even in antisense low-expressing cells that could not be identified by flow cytometry. In support of this, we observed that (i) antisense discrimination in the flow cytometry dot plots is more difficult than sense discrimination, (ii) activation agents like SAHA or PMA/Ionomycin invariably increase the frequency of antisense-expressing cells (6-fold) more than that of sense-expressing cells (1.2-fold; data not shown), (iii) anti-AS LNA transfection reduced the frequency of antisense-expressing cells by 0.4%, whereas gained that of sense-expressing cells by as much as 3% (**Figure 3**), and (iv) a part of the expanded AS+ cell clones lost antisense transcripts (Supplementary Figure S2) and hardly expressed sense transcripts even in the presence of SAHA or PMA/ionomycin (**Figure 5**). However, the present data do not offer sufficient resolution to define a threshold for the suppressive antisense transcript function. Furthermore, it is yet unclear why the cell clones established in this study have such different transcription patterns. This might be affected by differences in integration sites and/or the epigenetic status of the proviruses, the latter of which has already been suggested from us and others (Saayman et al., 2014; Chen et al., 2017; Zapata et al., 2017). An effect on antisense transcription by a strong host promoter activity can also not be excluded.

Antisense transcript production in latently infected T cells curtails latency reversal and thus may contribute to the observed inefficiency to reactivate HIV within infected individuals. However, such a mechanism is expected to account only for few such events. Extrapolating from our model system with fluorescent indicator gene expression, we estimate the frequency of latent AS+ cells to be about 1% of the total pool of latent HIV-bearing cells. This one percent may in chronically infected and long-term treated individuals amount to a critical barrier for eradication strategies that attempt to reactivate latent virus. However, while the antisense-mediated shut-off of virus production and reactivation is conceptually important, its clinical relevance is totally unknown. Nonetheless, the novel sense and antisense indicator system of rfl-HIV as used here for the characterization of individual latently infected cells may readily be used as a screening tool for compounds that would alter the antisense to sense transcription ratio of HIV. If such a regulatory switch could be achieved, it could reinforce therapeutic efforts in two directions: First, HIV antisense protein-targeting cytotoxic T cell strategies may become feasible and one could aim to eliminate virus-infected cells without ever producing virus progeny. Such a therapeutic strategy is under investigation in HTLV-I-infected patients with adult T cell leukemia (Sugata et al., 2015). Unfortunately, the rather low level of antisense transcription in HIV as compared to HTLV (Laverdure et al., 2016) has so far hampered efforts in this direction. Second, by enhancing the antisense transcription one may be able to lock HIV latency and thus keep the virus dormant and devoid of detrimental effects for the immune system. Indeed, such a concept fits well with a recently proposed block- and -lock strategy (Gavegnano et al., 2017; Jean et al., 2017; Kessing et al., 2017). By developing such latency-promoting agents in future, we might be able to achieve a genome status in which HIV-1 is recognized just as one amongst the many endogenous retroviruses.

## Conclusion

In conclusion, with the distinction of HIV antisense transcript positive and antisense transcript negative latently infected T lymphocytes, and the description of their functional differences with respect to latency reversing agents, we here add a new level of complexity to HIV latency. Whether this new knowledge can be turned into a new therapeutic strategy against HIV, however, has yet to be determined.

## Ethics Statement

Healthy, HIV-uninfected volunteers were included for donating blood samples. Blood was taken from these donors after explaining our experiments and obtaining an informed consent. This project was approved by the Institute of Medical Sciences, the University of Tokyo (IMSUT) Human Genome Research Ethics Committee and the Ethical Committee for the medical research using human resources in the National Institute of Infectious Diseases (NIID) Japan for the Use of Human Subjects.

## Author Contributions

MK-I and YT-Y: Study design. MK-I, JM, and YT-Y: Data curation. MK-I, JM, and RI: Acquisition of data. MK-I, KT, JM, and YT-Y: Analysis and interpretation of data. MK-I, KT, MY, TW, AM, and YT-Y: Validation. MK-I, KT, and JM: Writing the original manuscript. CB, MA, TW, AM, and YT-Y: Review and/or revision of the manuscript.

## Conflict of Interest Statement

The authors declare that the research was conducted in the absence of any commercial or financial relationships that could be construed as a potential conflict of interest.
