# Supplementary material for: HIV LTR-Driven Antisense RNA by Itself Has Regulatory Function and May Curtail Virus Reactivation From Latency
Source: Front Microbiol. 2018 May 25;9:1066. doi: 10.3389/fmicb.2018.01066 (PMC5980963; doi:10.3389/fmicb.2018.01066)
Supplement: Supplementary file 1 [file Data_Sheet_1.pdf]

Supplementary Material

HIV LTR-driven antisense RNA by itself has regulatory function and may curtail virus reactivation from latency

Mie Kobayashi-Ishihara[1,2], Kazutaka Terahara[1], Javier P Martinez[2], Makoto Yamagishi[3], Ryutaro Iwabuchi[1,4], Christian Brander[5,6,7], Manabu Ato[1,8], Toshiki Watanabe[9], Andreas Meyerhans[2,7], Yasuko Tsunetsugu-Yokota[1,2,10]

[1] Department of Immunology, National Institute of Infectious Diseases, Tokyo, Japan

[2] Infection Biology Group, Department of Experimental and Health Sciences, Universitat Pompeu Fabra, Barcelona, Spain

[3] Graduate School of Frontier Sciences, University of Tokyo, Tokyo, Japan

[4] Department of Life Science and Medical Bioscience, Waseda University, Tokyo, Japan

[5] IrsiCaixa – AIDS Research Institute, Badalona, Barcelona, Spain

[6] Universitat de Vic-Universitat Central de Catalunya (UVic-UCC), Vic, Barcelona, Spain

[7] Institució de Recerca i Estudis Avançats (ICREA), Barcelona, Spain

[8] Department of Mycobacteriology, Leprosy Research Center, National Institute of Infectious Diseases, Tokyo, Japan

[9] Department of Advanced Medical Innovation, St. Marianna University School of Medicine, Kawasaki, Japan

[10] Department of Medical Technology, School of Human Sciences, Tokyo University of Technology, Tokyo, Japan

*** Correspondence:**
Yasuko Tsunetsugu-Yokota, M.D., Ph.D.
yokotaysk@stf.teu.ac.jp

1.1 Supplementary Figures

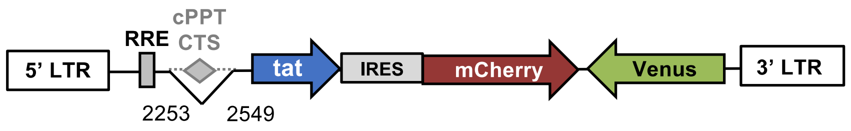

Supplementary Figure 1. ***Schematic description of* the *rfl-HIV proviral structure in DN1H11*.** A 300bp-deletion including cPPT/CTS region, which is involved in effective integration (Durand and Cimarelli, 2011), was found by sequencing analysis of the provirus in DN1H11 clone.

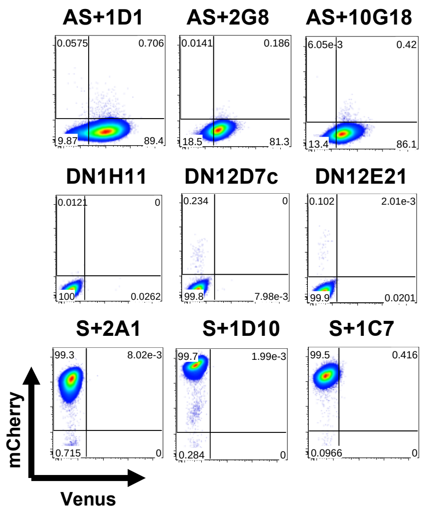

Supplementary Figure 2. ***Basal expression patterns of rfl-HIV-infected CEM clones.*** Upper, medium and bottom panels show representative dot plots of AS+, DN and S+ clones analyzed by flow cytometry, respectively.

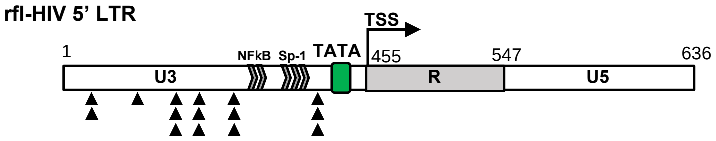

clone#	Nucleotide (base)					
	84	158	229	241	335	423
Original*	G	G	A	T	C	G
AS+1D1			G	G	T	C
AS+2G8	A		G	G	T	C
AS+10G18						
DN1H11						
DN12D7c	A		G	G	T	C
DN12E21						
S+2A1						
S+1C7						
S+1D10		A				
HXBII	A		G	G		C

*original nucleotides based on the sequence of rfl-HIV lentiviral vector
**empty means same nucleotide with the original one
***HXBII shows the nucleotides of HIV HXBII strain (GenBank#:K03455)

Supplementary Figure 3. ***Nucleotide structure of rfl-HIV 5'LTR in CEM clones.*** Upper panel shows positions and frequency of mutations mapped under rfl-HIV 5' LTR. TSS, Sense transcription start site; NF-kB, NF-kB binding sites; Sp-1, Sp-1 binding sites; TATA, TATA box for sense transcription. Lower panel shows the nucleotide information of mutated sites found in each clone.

1.2 Supplementary Reference

Durand, S., and A. Cimarelli. 2011. The inside out of lentiviral vectors. *Viruses* 3:132-159.
